# The joint effect of energy reduction with calcium supplementation on the risk factors of type 2 diabetes in the overweight population: a two-year randomized controlled trial

**DOI:** 10.18632/aging.202485

**Published:** 2021-02-11

**Authors:** Wei Wei, Wenbo Jiang, Wenbo Gu, Huanyu Wu, Haiyang Jiang, Guili Li, Qingrao Song, Jiaxin Huang, Xuanyang Wang, Lulu Wang, Changhao Sun, Tianshu Han, Ying Li

**Affiliations:** 1 Department of Nutrition and Food Hygiene, College of Public Health, Harbin Medical University, Harbin 150081, Heilongjiang Province, P. R. China; 2 Department of Postgraduate, The Third Affiliated Hospital of Harbin Medical University, Harbin 150081, Heilongjiang Province, P. R. China

**Keywords:** energy reduction, calcium supplementation, joint effect, RCT, T2DM

## Abstract

Both excessive energy intake and low calcium intake are inversely associated with the aging-related diseases, particularly for type 2 diabetes mellitus(T2DM). This study examined whether energy reduction coupled with calcium supplementation aided in the prevention of T2DM among the overweight population. A randomized controlled trial(RCT) of 1021 overweight participants was performed, in which participants were randomly assigned to 4 groups: 1) energy-reduction group(ERG), 2) calcium supplementation group(CSG), 3) energy-reduction with calcium supplementation group(ER-CSG), 4) control group(CG). Nutritional habits, anthropometric and diabetes-related indicators were measured at baseline and each follow-up time. To analyze the separate effects of dietary energy reduction and calcium supplementation, ERG and ER-CSG were integrated into ERGs. Similarly, CSG and ER-CSG were integrated into CSGs. Compared to the non-energy-reduction groups(NERGs), ERGs had lower values of ΔBMI(-0.9kg/m^2^), ΔFSG (-0.34mmol/L), ΔHbA1c(0.16%), and ΔHOMA-IR(-0.13), and higher value of ΔGutt index(-5.82). Compared to the non-calcium supplementation groups(NCSGs), the ΔGutt index(-5.46) in CSGs showed a significant decrease. Moreover, these risk factors for T2DM were most effectively ameliorated in ER-CSG group with the decreased values of ΔFSG(-0.42mmol/L), ΔGutt index(-0.73), and the slowest increasing rate value of Δ2h-glucose(0.37mmol/L). This RCT demonstrated that energy-reduction with calcium supplementation was a useful dietary intervention strategy for preventing the development of T2DM in the overweight population.

## INTRODUCTION

The prevalence of type 2 diabetes mellitus (T2DM) has become one of the most important global problems [1], and unhealthy dietary patterns have been recognized as the most important risk factors for T2DM [2, 3]. Previous studies reported that both excessive energy intake and deficient mineral intake were associated with the development of T2DM [4, 5], and the nutritional status of excessive energy intake coupling with insufficient mineral intake was frequently seen in the nutrition survey [6]. However, it is still largely unknown whether and how this dietary pattern would impact the development of T2DM.

As an essential macronutrient, dietary calcium intake has been known to play a crucial role in maintaining bone health [7]. In recent years, accumulating epidemiological studies have also shown that deficient dietary calcium intake is associated with a higher incidence of T2DM [8, 9]. Animal experiments demonstrated that intracellular calcium concentration could regulate both insulin secretion in β cells and insulin sensitivity in insulin-targeted tissues [10]. However, the results of RCT showed that calcium supplementation could not improve fasting serum glucose (FSG) and insulin resistance (IR) [11–13]. In the animal studies, we observed that compared to rats fed with a high-fat diet, the rats fed with a high-fat and calcium-deficient diet were more likely to have diabetes [14], which implied that the coexist of low calcium intake and high dietary energy intake was more easily to promote the development of T2DM than low calcium intake or high energy intake alone. In this study, we hypothesized that the dietary pattern of calcium supplementation with energy reduction could more effectively prevent the development of T2DM than calcium supplementation or energy reduction alone. To examine this hypothesis and provide more effective dietary strategies for preventing T2DM, a randomized controlled trial (RCT) was conducted to comprehensively evaluate the effect of the interaction between the dietary nutritional status of calcium and energy intakes on the risk factors for T2DM.

## RESULTS

### Baseline characteristics

**Figure 1:**
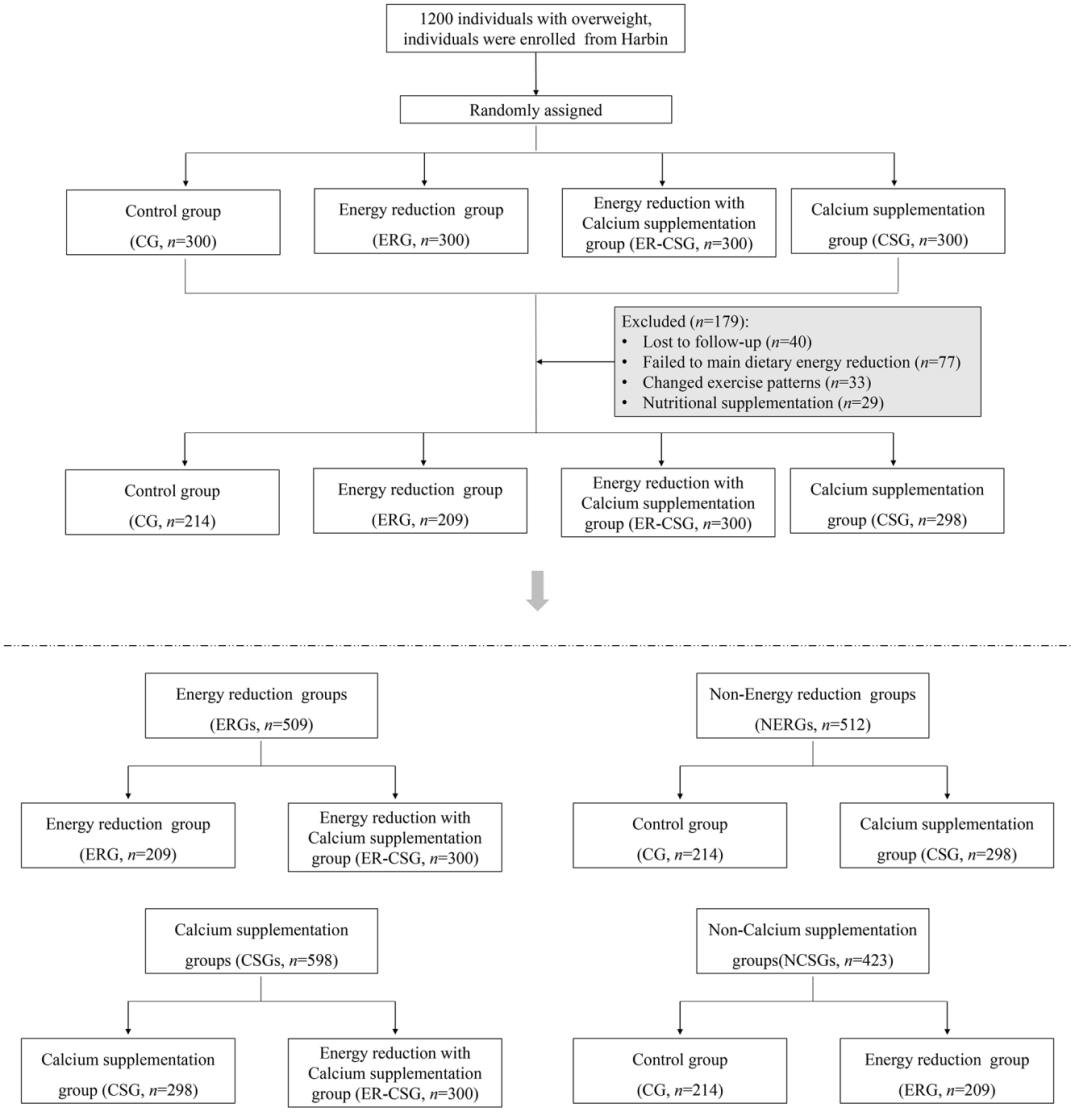
**The flow diagram of the RCT.**

After the intervention, 179 participants have been excluded due to loss of follow-up, poor compliance, and medications. A total of 1021 overweight participants with low dietary calcium intake were finally recruited, details are shown in the flow diagram Figure 1. Specifically, 1) 209 participants were in the energy-reduction group (ERG), which reduced the dietary energy intake while maintaining the original calcium intake, 2) 298 participants were in the calcium supplementation group (CSG), which maintained the original dietary energy intake while receiving calcium supplementation, 3) 300 participants were in the energy-reduction with calcium supplementation group (ER-CSG), which reduced the dietary energy intake and received calcium supplementation. And the 214 participants without dietary intervention were set as the control group (CG). Among them, 509 participants with reducing energy intake were assigned to the energy-reduction groups (ERGs), 512 participants without reducing energy intake were assigned to the non-energy-reduction groups (NERGs). In addition, 598 participants with calcium supplementation were assigned to the calcium supplementation groups (CSGs), and 423 participants without calcium supplementation were assigned to the non-calcium supplementation groups (NCSGs).

The characteristics of these study variables at baseline are presented in Tables 1, 2. No significant different dietary intakes of energy, calcium and serum 25(OH)D_3_ were observed among the groups that were compared.

**Table 1:** The baseline characteristics in ERGs, NERGs, CSGs, and NCSGs.

**Variables**	**Dietary energy reduction**		**Calcium supplementation**	
	**ERGs**	**NERGs**	***P*-value**	**CSGs**	**NCSGs**	***P*-value**
Age (years)	51.4 (9.4)	49.0 (8.9)	0.011	50.1 (9.4)	50.1 (9.2)	0.934
Male [n (%)]	232 (45.5)	212 (41.5)	0.433	221 (36.9)	251 (59.3)	<0.001
Smoking [n (%)]	94 (18.4)	97 (19.0)	0.900	88 (14.8)	121 (28.6)	0.004
Drinking [n (%)]	168 (33.0)	212 (41.4)	0.264	211 (35.3)	190 (45.0)	0.219
Regular exercise [n (%)]	192 (37.8)	259 (50.5)	0.011	247 (41.3)	217 (51.2)	0.067
Energy intake (kcal/d)	2165 (984)	2076 (774)	0.529	2116 (931)	2261 (943)	0.150
Ca_dietary (mg/d)	549 (285)	551 (279)	0.961	544 (280)	565 (285)	0.481
Ca^2+^_serum (mmol/L)	2.24 (0.01)	2.27 (0.01)	0.102	2.25 (0.01)	2.26 (0.01)	0.449
25(OH)D_3_ (ng/ml)	17.26(4.33)	16.84(4.04)	0.300	16.91(3.76)	17.38(5.08)	0.353
BMI (kg/m²)	27.7 (0.18)	27.4 (0.18)	0.295	27.5 (0.15)	27.6 (0.23)	0.740
FSG (mmol/L)	4.79 (0.06)	4.57 (0.06)	0.010	4.67 (0.05)	4.74 (0.08)	0.453
2h-glucose (mmol/L)	6.13 (0.11)	6.20 (0.11)	0.671	6.10 (0.09)	6.36 (0.14)	0.120
Fasting insulin (μ U/L)	9.52 (0.44)	9.31 (0.46)	0.744	9.69 (0.36)	8.81 (0.55)	0.190
2h-insulin (μ U/L)	54.5 (3.5)	53.5 (3.6)	0.846	54.3 (2.8)	53.4 (4.3)	0.863
HbA1c (%)	5.56 (0.03)	5.60 (0.03)	0.394	5.61 (0.03)	5.51 (0.04)	0.071

Note: Data are presented as mean (Standard deviation) with adjustment for age and gender.

**Table 2:** The baseline characteristics in ER-CSG, CSG, ERG, and CG.

**Variables**	**ER-CSG**	**CSG**	**ERG**	**CG**	**P-value**
Age (years)	52.3 (0.77)	48.0 (0.73)	50.1 (1.1)	50.1 (1.2)	0.001
Male [n (%)]	110 (36.6)	111 (37.2)	178 (64.2)	125 (53.6)	<0.001
Smoking [n (%)]	42 (14.1)	46 (15.4)	75 (26.9)	67 (28.6)	<0.001
Drinking [n (%)]	93 (31.0)	109 (36.5)	104 (37.3)	104 (44.6)	<0.001
Regular exercise [n (%)]	101 (33.8)	143 (48.1)	129 (46.3)	134 (57.1)	<0.001
Energy intake (kcal/d)	2102 (82)	2076 (79)	2039 (112)	2307 (133)	0.426
Ca_dietary (mg/d)	571 (313)	519 (245)	504 (211)	539 (345)	0.157
Ca2+_serum (mmol/L)	2.23 (0.01)	2.28 (0.02)	2.28 (0.02)	2.24 (0.02)	0.002
25(OH)D3 (ng/ml)	16.62(4.57)	16.47(2.77)	16.99(3.78)	17.85(6.30)	0.113
BMI (kg/m²)	27.6 (0.23)	27.4 (0.22)	27.6 (0.32)	28.0 (0.38)	0.664
FSG (mmol/L)	4.72 (0.07)	4.62 (0.07)	4.95 (0.10)	4.42 (0.12)	0.005
2h glucose (mmol/L)	6.14 (0.13)	6.18 (0.13)	6.10 (0.19)	6.29 (0.22)	0.923
Fasting insulin (μ U/L)	9.23 (0.51)	10.19 (0.49)	9.94 (0.70)	7.10 (0.83)	0.012
2h insulin (μ U/L)	55.3 (4.12)	55.5 (3.92)	51.8 (5.69)	54.9 (6.76)	0.964
HbA1c (%)	5.62 (0.04)	5.59 (0.04)	5.44 (0.06)	5.61 (0.07)	0.067

Note: Data are presented as mean (Standard deviation) with adjustment for age and gender.

### Compliance assessment

**Figure 2:**
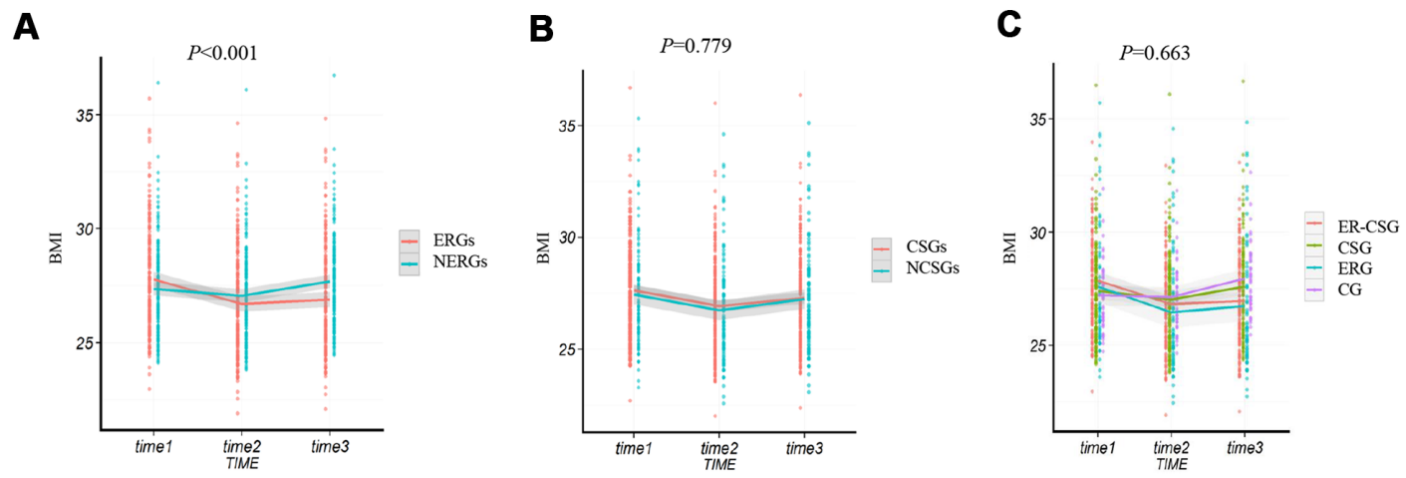
**The effect of energy reduction and calcium supplementation on BMI adjusted for age, gender, smoking, drinking, and regular exercise.** (**A**) Comparison of BMI changes between ERGs and NERGs. (**B**) Comparison of BMI changes between CSGs and NCSGs. (**C**) Comparison of BMI changes among CG, ERG, CSG, and ER-CSG. Note: *P*-value for the difference in the joint effect of intervention and time.

After the dietary energy restriction, the dietary alteration in the ERGs and NERGs are presented in Supplementary Table 1. In comparison to the dietary information at baseline, participants in the ERGs had lower intakes of rice, wheat, potato, beans, livestock, poultry, eggs, snack, and micronutrients (carbohydrate, protein, lipid), while had higher intakes of vegetables and fruits. The ERGs had an average decreased energy intake of 492 kcal/d (23%) after the 2-year intervention. However, compared to baseline, the NERGs had higher intakes of wheat, potato, beans, livestock, poultry, fish, snack, carbohydrate, and had an average increased energy intake of 106 kcal/d (5%) after the intervention. After the 2 years dietary intervention, the ΔBMI (body mass index) in the ERGs showed a significant decrease (-0.9 kg/m^2^, *P* <0.001, Figure 2A), compared to the NERGs (0.3 kg/m^2^). Whereas, no significant difference of ΔBMI was observed between the CSGs and NCSGs (-0.3 kg/m^2^ vs -0.2 kg/m^2^, *P*=0.779, Figure 2B). In addition, the ΔBMI in the CG, ERG, CSG, and ER-CSG was 0.7 kg/m^2^, -0.9kg/m^2^, 0.2 kg/m^2^ and -1kg/m^2^, respectively. The four intervention models had no significant difference in ΔBMI (*P*=0.663, Figure 2C).

**Figure 3:**
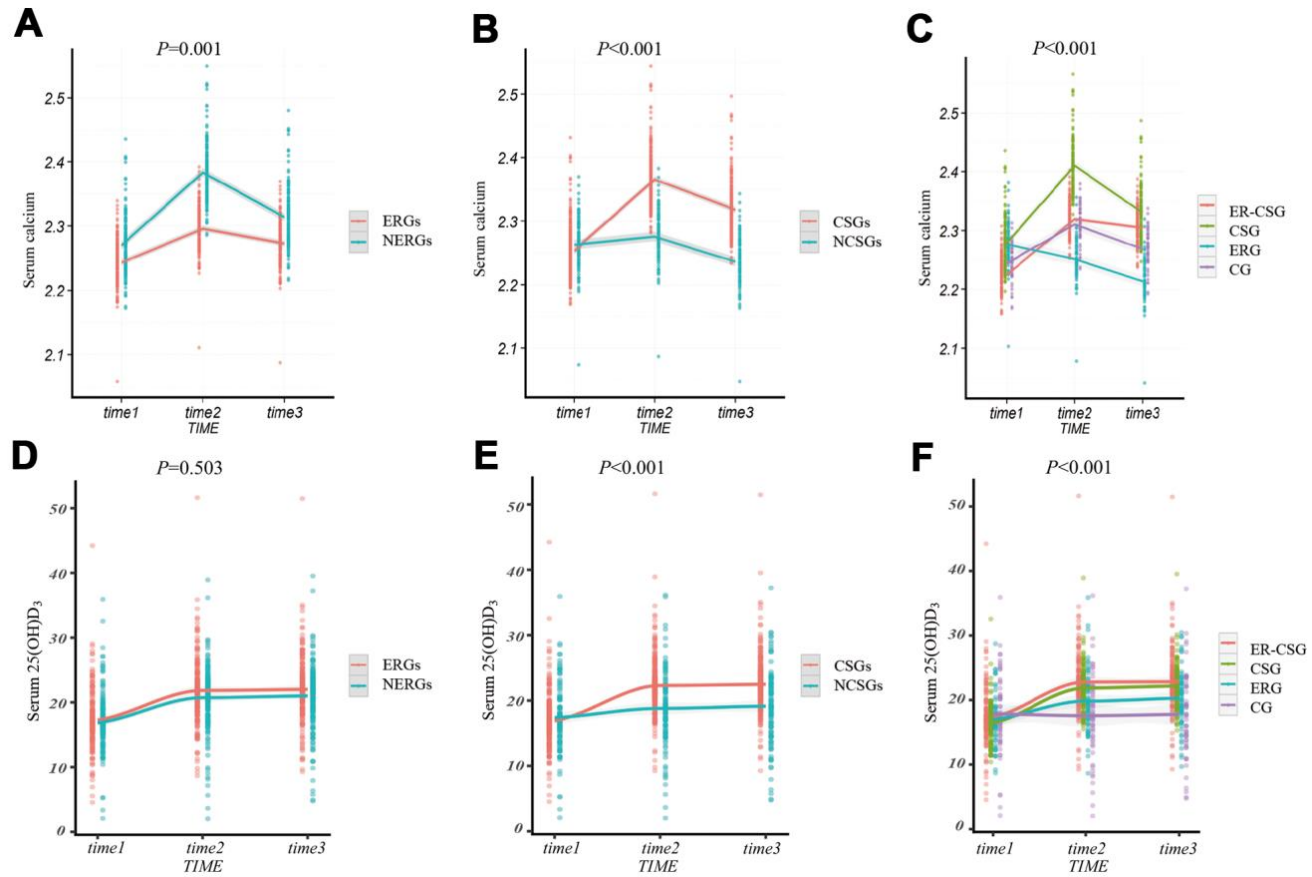
**The effect of energy reduction and calcium supplementation on serum calcium, serum 25(OH)D_3_ adjusted for age, gender, smoking, drinking, regular exercise, and BMI.** (**A**) Comparison of serum calcium changes between ERGs and NERGs. (**B**) Comparison of serum calcium changes between CSGs and NCSGs. (**C**) Comparison of serum calcium changes among CG, ERG, CSG, and ER-CSG. (**D**) Comparison of serum 25(OH)D_3_ changes between ERGs and NERGs. (**E**) Comparison of serum 25(OH)D_3_ changes between CSGs and NCSGs. (**F**) Comparison of serum 25(OH)D_3_ changes among CG, ERG, CSG, and ER-CSG. Note: *P*-value for the difference in the joint effect of intervention and time.

After adjustments for confounding factors, participants in the ERGs had a lower Δserum calcium than participants in NERGs (0.03 mmol/L vs 0.04 mmol/L, *P*=0.001, Figure 3A). The Δserum calcium in the CSGs displayed a significant increase, compared to that in the NCSGs (0.07 mmol/L vs -0.02 mmol/L, *P*<0.001, Figure 3B). We also found that the ER-CSG had the highest Δserum calcium value (0.08 mmol/L, all the *P*<0.001, Figure 3C) in the four groups. In addition, the Δserum 25(OH)D_3_ level was not significantly different between the NERGs and ERGs (4.17 ng/ml vs 4.76 ng/ml, *P*=0.503, Figure 3D); and compared to the NCSGs, the Δserum 25(OH)D_3_ in the CSGs was significantly increased (1.76 ng/ml vs 5.58 ng/ml, *P*<0.001, Figure 3E). Besides, the ER-CSG had a higher Δserum 25(OH)D_3_ (6.23 ng/ml, Figure 3F), compared to the other three groups (all the *P<*0.001).

### Effect on ΔFSG and Δ2h-glucose

**Figure 4:**
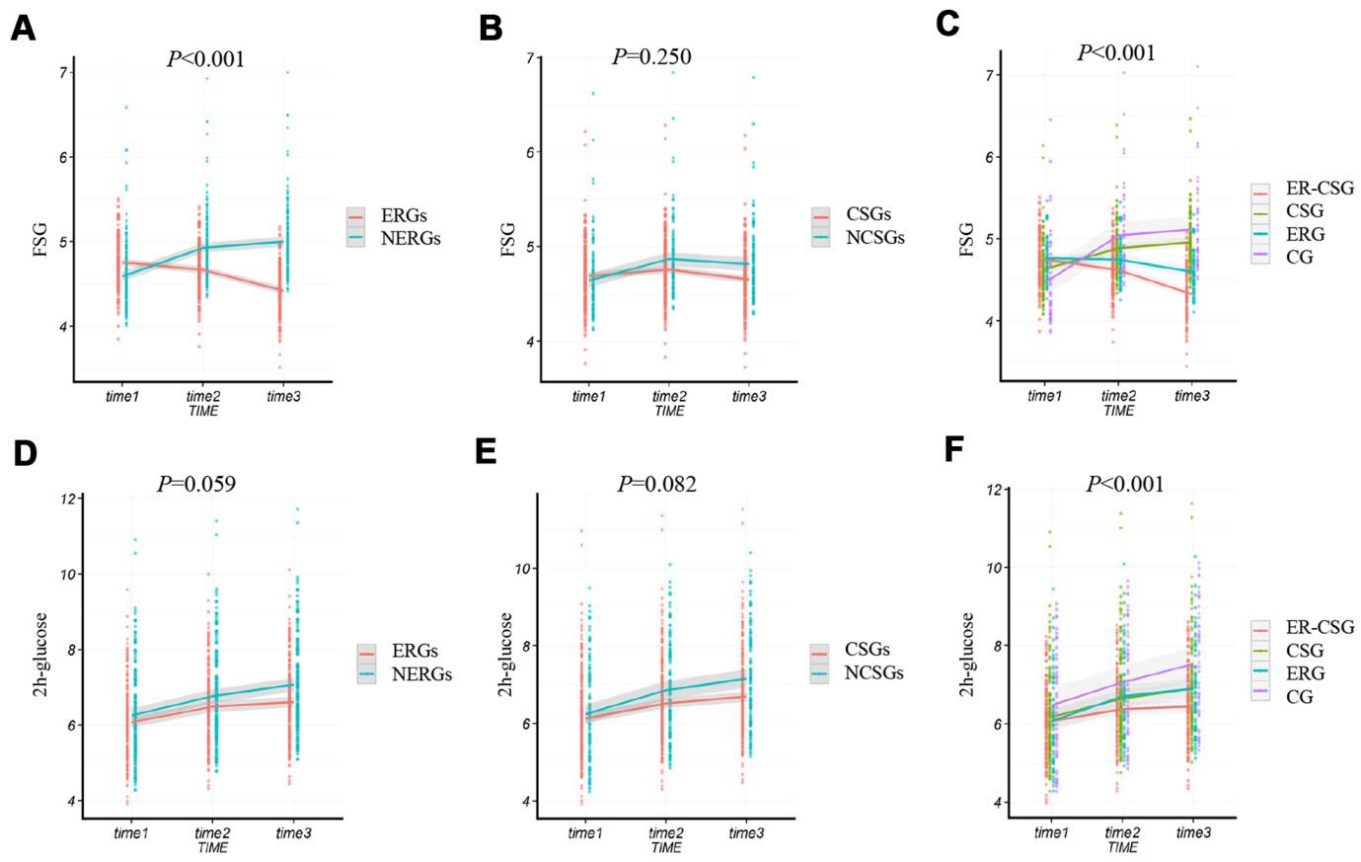
**The effect of energy reduction and calcium supplementation on FSG, and 2h-glucose adjusted for age, gender, smoking, drinking, regular exercise, and BMI.** (**A**) Comparison of FSG changes between ERGs and NERGs. (**B**) Comparison of FSG changes between CSGs and NCSGs. (**C**) Comparison of FSG changes among CG, ERG, CSG, and ER-CSG. (**D**) Comparison of 2h-glucose changes between ERGs and NERGs. (**E**) Comparison of 2h-glucose changes between CSGs and NCSGs. (**F**) Comparison of 2h-glucose changes among CG, ERG, CSG, and ER-CSG. Note: *P*-value for the difference in the joint effect of intervention and time.

In this study, we found that the ΔFSG in the ERGs had a lower value than NERGs (-0.34 mmol/L vs 0.41 mmol/L, *P*<0.001, Figure 4A). Whereas, the result showed there was a non-significant difference in the ΔFSG between the CSGs and NCSGs (-0.04 mmol/L vs 0.17 mmol/L, *P*=0.250, Figure 4B). Moreover, no significant difference in the Δ2h-glucose (2 hours post-load serum glucose) was also observed either between the ERGs and NERGs (0.53 mmol/L vs 0.81 mmol/L, *P*=0.059, Figure 4D) or between the CSGs and NCSGs (0.56 mmol/L vs 0.92 mmol/L, *P*=0.082, Figure 4E).

Compared to the other three groups, the ER-CSG had a higher reduction in the ΔFSG (-0.42 mmol/L, all the *P* <0.001, Figure 4C). Although the 2h-glucose presented an upward trend, participants in the ER-CSG had the lowest increase in Δ2h-glucose among all the groups (0.37 mmol/L, all the *P* <0.001, Figure 4F).

### Effect on HOMA-IR and ΔGutt index

**Figure 5:**
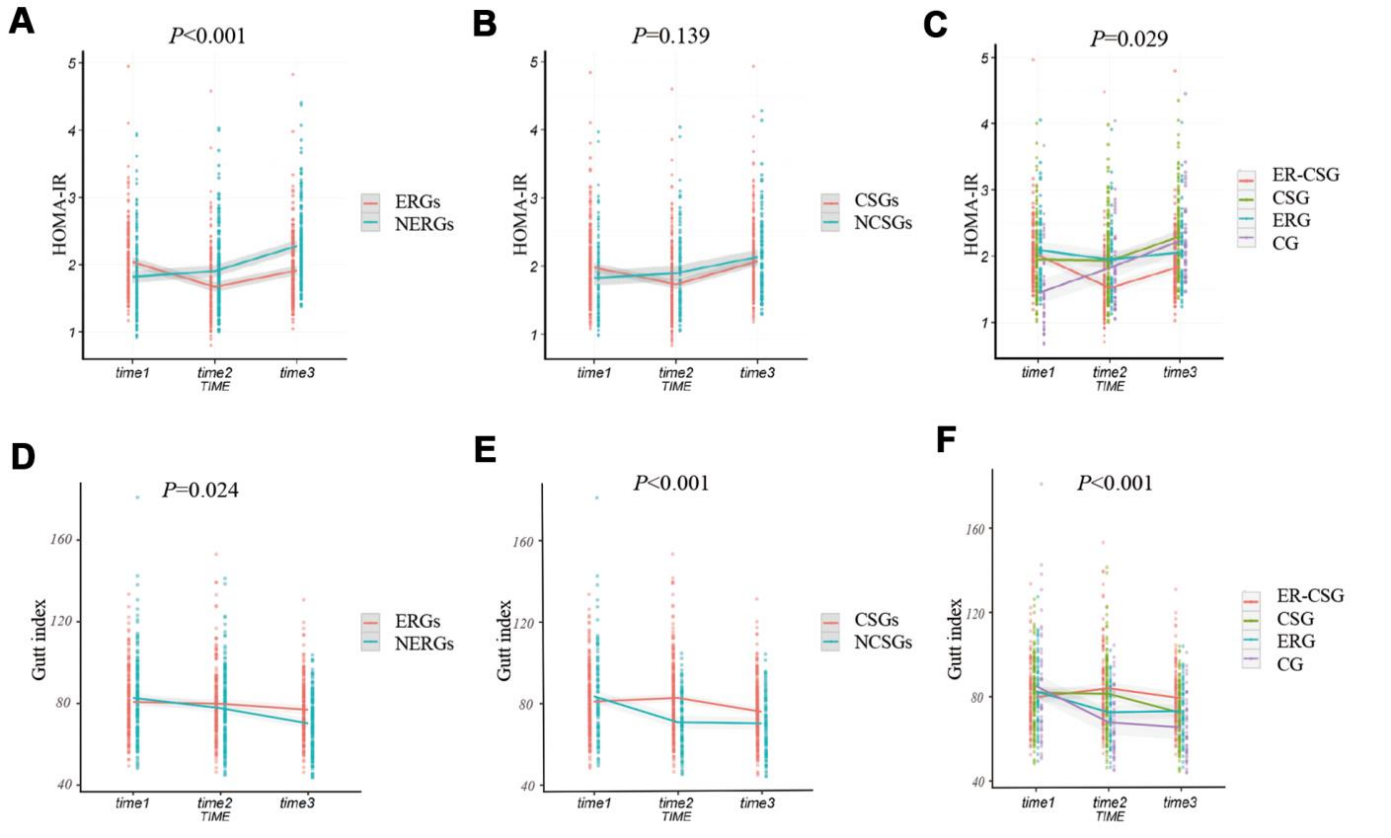
**The effect of energy reduction and calcium supplementation on 2h-insulin, and HOMA-IR adjusted for age, gender, smoking, drinking, regular exercise, and BMI.** (**A**) Comparison of HOMA-IR changes between ERGs and NERGs. (**B**) Comparison of HOMA-IR changes between CSGs and NCSGs. (**C**) Comparison of HOMA-IR changes among CG, ERG, CSG, and ER-CSG. (**D**) Comparison of Gutt index changes between ERGs and NERGs. (**E**) Comparison of Gutt index changes between CSGs and NCSGs. (**F**) Comparison of Gutt index changes among CG, ERG, CSG, and ER-CSG. Note: *P*-value for the difference in the joint effect of intervention and time.

The ERGs had a significantly lower ΔHOMA-IR (Homeostasis assessment model for insulin resistance) than the NERGs (-0.13 vs 1, *P*<0.001, Figure 5A). Whereas, the ΔHOMA-IR value showed no difference between participants in the CSGs and NCSGs (0.09 vs 0.31, *P*=0.139, Figure 5B). Furthermore, we analyzed the ΔHOMA-IR level in the four subgroups. The result also exhibited a non-significant different decrease in ΔHOMA-IR among the four groups (ER-CSG: -0.17, CSG: 0.34, ERG: -0.03, CG:0.79, *P*=0.029, Figure 5C).

Participants in the ERGs had a higher ΔGutt index than participants in the NERGs(-5.82 vs -10.48, *P*=0.024, Figure 5D). Similarly, the participants in the CSGs also presented a significantly higher ΔGutt index, compared to the participants in the NCSGs (-5.46 vs -13.75, *P*<0.001, Figure 5E). Besides, the ER-CSG had a higher ΔGutt index, when comparing to the other groups (-0.73, *P*<0.001, Figure 5F).

### Effect on ΔHbA1c

**Figure 6:**
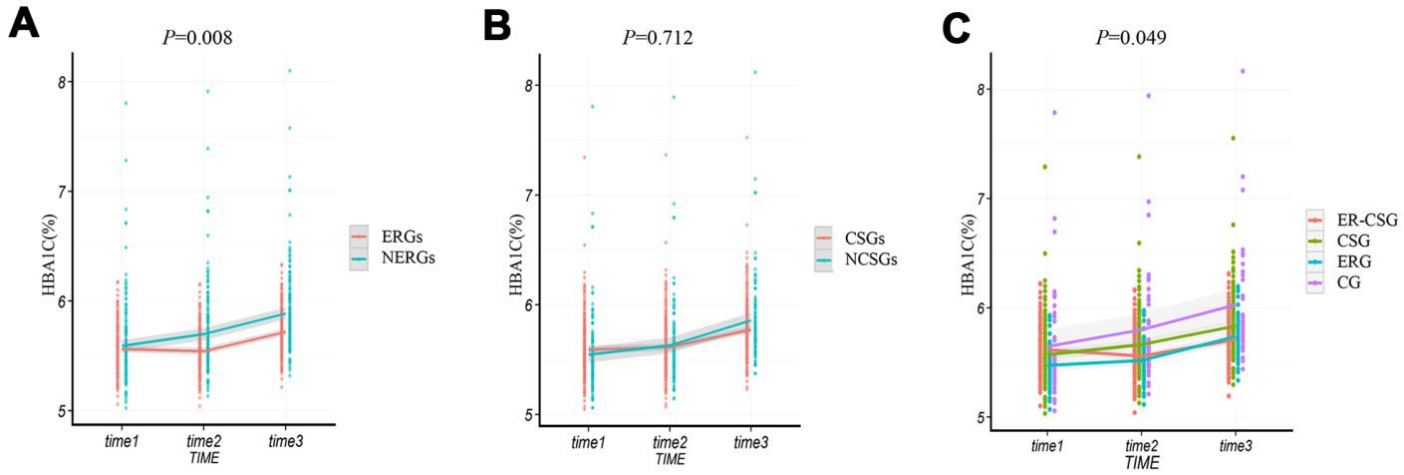
**The effect of energy reduction and calcium supplementation on HbA1c adjusted for age, gender, smoking, drinking, regular exercise, and BMI.** (**A**) Comparison of HbA1c changes between ERGs and NERGs. (**B**) Comparison of HbA1c changes between CSGs and NCSGs. (**C**) Comparison of HbA1c changes among CG, ERG, CSG, and ER-CSG. Note: *P*-value for the difference in the joint effect of intervention and time.

Similar to the results of Δ2h-glucose, the averages of ΔHbA1c (glycated hemoglobin) in all groups had increasing trends. And the ERGs had a lower upward value of ΔHbA1c than the NERGs (0.16% vs 0.30%, *P*=0.008, Figure 6A). However, no significant difference in ΔHbA1c level was found between the CSGs and NCSGs (0.18% vs 0.32%, *P*=0.712, Figure 6B). And non-significant difference in ΔHbA1c was also observed among the four groups (ER-CSG: 0.10%, CSG: 0.26%, ERG: 0.27%, CG: 0.38%, *P*=0.049, Figure 6C).

## DISCUSSION

Although a few epidemiological studies have shown that the dietary pattern of excess energy and low calcium intakes is associated with increased risks of obesity and diabetes, there are still no RCTs to clarify the causal relationship between this dietary pattern and T2DM. In this study, an RCT including 1021 overweight participants was conducted to examine whether energy reduction and calcium supplementation had a joint effect on the development of diabetes. Based on the 2 years intervention, the participants with energy reduction had a significantly lower dietary energy intake, and participants with calcium supplementation had a significantly higher serum calcium concentration and serum 25(OH)D_3_, which indicated that the interventions were effective.

This study not only found that ERGs had a lower increased Δserum calcium concentration than the NERGs but also observed that the ER-CSG had the highest Δserum calcium and Δserum 25(OH)D_3_ levels among the four groups. Previous evidence has documented that obese rats with dietary energy reduction had a lower serum calcium concentration and lower expression levels of calcium-regulated-dependent protein kinase, as well as higher intracellular calcium level [15], which supported our result that energy reduction could regulate the serum calcium. For the participants in the ER-CSG, although energy reduction could reduce the serum calcium, calcium supplementation might still enhance the serum calcium level. Also, lower serum calcium caused by energy reduction probably could promote the dietary calcium absorption of VD supplementation, which could further stimulate the increase of serum calcium. The above points might be the reasons for the greater increase in serum 25(OH)D_3_ and serum calcium in the ER-CSG. The results illustrated that energy reduction and calcium supplementation had a joint effect on regulating serum calcium concentration.

Excess dietary energy intake is well-recognized as the main risk factor for T2DM. Evidence has shown that excess dietary energy intake could result in obesity and T2DM [16]. Our results showed the average ΔBMI in the ERGs had a greater decrease than ERGs, indicating that energy reduction could promote weight loss. Also, energy reduction significantly reduced the fasting glucose, hepatic and peripheral IR, which were consistent with previous researches [17–20]. In mechanism, the improvement of these indicators is probably caused by the reduction of body fat and the increased ability of the peripheral insulin-targeted tissue. It was documented that a lower dietary energy intake could reduce the level of serum very-low-density lipoproteins (VLDL), and improve the ectopic accumulation of peri-hepatic fat and lipotoxicity [21]. Accumulating evidence also illustrated that energy reduction could increase the serum adiponectin level and affinities of the insulin receptor, as well as improve insulin sensitivity [22, 23]. Although some studies showed that energy reduction could reduce the area under the curve of serum glucose in the oral glucose tolerance test [24], a marginal difference of Δ2h-glucose level was observed between the ERGs and NERGs in this study. It has been reported that the 2h-glucose level reflects insulin sensitivity, especially for the utilized ability of insulin-targeted tissues [25]. Of note, the insulin-targeted tissue is mainly composed of fat and skeletal muscle, which have a strong correlation with BMI. And our study adopted a lower intensity energy reduction according to the real-life conditions rather than greatly reducing the dietary energy intake (32%-52%) in other studies [17–19]. Therefore, the decreased BMI level was limited, leading to a less improvement effect of peripheral IR. This might be the underlying mechanism of non-significant change of 2h-glucose.

A few epidemiological studies have found a negative association between dietary calcium intake and T2DM [9, 26]; however, the results of RCTs regarding calcium supplement intervention frequently cannot validate this beneficial effect [11–13, 27]. In line with these RCTs, our study also showed calcium supplementation only increased the serum calcium concentration and increased the peripheral insulin sensitivity. It has been reported that a low dietary calcium intake could increase the intracellular calcium level, and further impair the transduction of insulin signal [28–30]. The reason for the improvement of peripheral insulin sensitivity might be due to that the calcium supplementation restores the damage of insulin signal transduction caused by dietary calcium-deficiency. Given ΔHOMA-IR in the CSGs was not statistically different from NCSGs, suggesting that calcium supplementation probably could not improve hepatic IR. Moreover, the result of the ΔFSG and Δ2h-glucose also indicated that calcium supplementation alone probably could not effectively prevent the development of T2DM in participants at high risk of diabetes. Further, compared to the other 3 type intervention modes, energy reduction with calcium supplementation showed significantly lower ΔFSG, Δ2h-glucose, and higher ΔGutt index, suggesting that energy reduction combined with calcium supplementation had the joint effect on improving the serum glucose. The above results indicated that energy reduction with calcium reduction could not only reduce the abnormal accumulation of fat but also increase the affinity of peripheral insulin receptors of fat and skeletal muscle. Therefore, these two aspects may be the potential mechanisms underlying this synergistic ameliorated effect on serum glucose.

### Strength and limitation

This study was the first long-term RCT that demonstrated energy reduction with calcium supplementation could synergistically improve the risk factors for T2DM, providing a more effective dietary intervention strategy for overweight participants. However, we also recognized that this study had certain limitations. Firstly, we did not measure the levels of insulin and the ratio of insulin and glucose at 30 minutes of OGTT, which could more accurately reflect the insulin secretion ability of β cells [31]. Hence, the joint effect of energy restriction and calcium supplementation on insulin secretory should be further explored. And the underlying molecular mechanism of the joint effect is warranted to be confirmed. Secondly, unlike the ERG, other groups maintained the original dietary pattern or regularly received new calcium tablets each month through a face-to-face way; it was difficult for the participants to persist in the energy restriction diet without strict supervision in the ERG. Therefore, the number of people lost in follow-up in the ERG was higher. In order to ensure that participants have a better adherence, we adopted a gentle energy restriction dietary intervention based on the original eating habits and supported the participants to choose food in their own way. Also, we conducted monthly follow-up visits by phone to improve the adherence of participants in the ERG. However, the number of people lost in follow-up in the ERG was still relatively high. Although the final number of individuals in the ERG met the minimum sample size, future studies are still necessary to conduct more effective measures to control the number of people lost in follow-up in the energy-restricted RCTs.

In conclusion, this study for the first time demonstrated that energy reduction with calcium supplementation in the overweight population could more effectively reduce the risk factors for T2DM than energy reduction or calcium supplementation alone.

## MATERIALS AND METHODS

### Study population

The participants were approved to participate if they (1) were between 20 and 70 years old, (2) had been living in Harbin for at least 10 years, (3) were without major diseases, such as cancer or type 1 diabetes mellitus, (4) were without oral hypoglycemic drugs, insulin injections, and losing weight, (5) were with dietary calcium intake less than recommendations (Adequate intake,<800 mg/d), and no calcium or vitamin D supplementation at least for six months, (6) were overweight (body mass index, BMI ≥24kg/m²). A total of 1200 participants were recruited in this study. The approval of Ethics Committee of Harbin Medical University and written informed consent of all participants were obtained before this RCT. This RCT was registered at http://www.chictr.org.cn (ChiCTR-TRC-12002829).

### Questionnaire survey and anthropometric measurements

A structured questionnaire was used to collect the detailed information for the participants, including age, sex, dietary habits, disease histories, medication use, current smoker and drinker, and physical activity. Food Frequency Questionnaire (FFQ) was conducted to measure the frequency and quantity of food intake in the past year. Daily dietary intake of energy and nutrients were calculated based on the Chinese Food Composition Table (2004).

The anthropometric characteristics were obtained by well-trained personnel of Community Health Centers, such as height (m), weight (kg), BMI (kg/m^2^), waist circumference (cm), hips circumference (cm), systolic and diastolic blood pressure (mm/Hg). BMI was calculated based on the equation: BMI (kg/m^2^) = weight (kg)/ height^2^ (m^2^). And after a 10-minute rest in a sitting position, blood pressures were measured 3 times from 8: 00 AM to 10:00 AM by a standard mercury sphygmomanometer on the right arm, and the average value of each individual was used for further analysis.

In addition, an oral glucose tolerance test (OGTT) was also conducted on all participants according to the World Health Organization guidelines. Blood samples (before and after OGTT) were collected and divided into two parts. One part was used to measure the HbA1c (%). The other part was centrifuged at 3000 rpm for 15 min, and the upper serum of each sample was transferred into a new tube for further measurements of FSG (mmol/L) and fasting serum insulin (μ U/L), serum Ca^2+^(mmol/L), serum 25(OH)D_3_ (ng/ml), serum 2h-glucose (mmol/L) and serum 2h-insulin (μ U/L). FSG, serum 2h-glucose, and serum Ca^2+^ were measured by an automatic biochemistry analyzer (7100; Hitachi, Tokyo, Japan). Fasting insulin and serum 2h-insulin were measured using the immunofluorescence analysis (Tosoh automated enzyme immunoassay analyzer AIA-2000ST). The blood HbA1c (%) was measured by ARKRAY HA-8380 automatic glycosylated hemoglobin analyzer (Tokyo, Japan). Serum 25(OH)D_3_ was examined by Ultra high-performance liquid chromatography coupled with triple quadrupole mass spectrometry (UPLC/XEVO TQ MS, Waters ACQUITY UPLC Coupling Waters Xevo) [32]. Moreover, the Gutt index was calculated as an indicator of peripheral insulin resistance, which is based on glucose uptake rates, metabolic clearance rates, and mean serum insulin by the following equation: [75 000+(FSG−2-h glucose)×0.19×body weight]/(120×log [(fasting insulin+2-h insulin)/2]×[(FSG glucose+2-h glucose)/2]). And the HOMA-IR calculator https://www.dtu.ox.ac.uk/homacalculator/ (developed by the University of Oxford in 2004) was employed to estimate the hepatic insulin resistance level.

### Randomization and intervention strategies

1200 participants enrolled in this study were randomly assigned (1:3) by a statistician as follows. All participants were matched to other participants based on BMI and dietary intake of energy and calcium. The first participant in each matched pair was randomly assigned to either group. The matched 3 participants for each pair were automatically assigned to the other 3 treatment groups. These four groups were defined as CG, ERG, CSG, and ER-CSG, respectively. To ensure the homogeneity of outcome variables at baseline between the 4 groups, the BMI, energy intake, and dietary calcium intake at baseline were tested. If the homogeneity of the outcome variables at baseline was not satisfied, the participants were randomly assigned again.

The dietary energy reduction was adjusted by nutrition education. To enable the participants to realize the association between lower energy intake and health, and master the method for adjusting their dietary energy intake, we adopted 3 ways: 1) conducted lectures on dietary energy and health, 2) distributed dietary guide books and compact disc, such as "Chinese Residents Dietary Guidelines", and "Chinese Food Nutrition Table (2004)", 3) recommended nutrition and health-related educational websites. Participants should master the method of food energy calculation, and reduce the intake of high energy density food by substituting it with fruits and vegetables. Diets were structured to provide a healthy and balanced level of macronutrients: 20-30% calories from fat, 10-12% calories from proteins, and 55-65% calories from carbohydrates.

The participants with calcium supplementation were received 400mg calcium and 125UI vitamin D_3_ per day, and they were instructed to take after breakfast. They used empty bottles to obtain new calcium tablets every month. The placebo was given to participants without calcium supplementation. The entire intervention period is 2 years (from 2013/01/01 to 2015/12/31).

Dietary information of each subject was reviewed every 3 months to ensure dietary adherence during the intervention time.

### Follow-up indicators

To ensure the implementation of the intervention, we conducted supervision during the whole RCT period. The dietary intake and biochemical indicators related to T2DM were followed up once a year, including BMI (kg/m^2^), waist circumference (cm), hips circumference (cm), systolic and diastolic blood pressure (mm/Hg), FSG (mmol/L), fasting serum insulin (μ U/L), blood HbA1c (%), serum Ca^2+^(mmol/L), serum 25(OH)D_3_ (ng/ml), serum 2-glucose (mmol/L), serum 2-insulin (μ U/L), Gutt index and HOMA-IR.

### Statistical Analysis

The sample size was determined by the change in FSG, which were as the primary outcome measure. At a 5% significance level and with 90% statistical power, a sample size of 132 per group was required to detect a 1.0 mmol/L difference in FSG with an SD of 2.5 mmol/L (18). Allowing for a 15% drop-out rate, at least 156 subjects needed to be enrolled per group.

Demographic characteristics, anthropometric measurements, dietary energy, and nutrient intakes were presented as mean (SD) for continuous variables or number percentage for categorical variables. The change rates (Δ) in dietary intakes of energy and nutrients, and anthropometric measurements were also exhibited as mean (SD). And the change rates of the indicators were computed by mean difference (after intervention-before intervention). Generalized linear models adjusted for age were conducted to compare continuous variables in baseline characteristics between the 4 groups. Age, sex, smoking, drinking, physical activity, and BMI were set as non-dietary covariates in this study. A mixed linear model was established to analyze the effect of the different interventions on the change rates in indicators with fixed and random effects. Participants are random-effects, and the intervention modes are fixed-effects. The statistical significance was presented by the P interaction between the intervention mode and time.

All statistical analyses were performed using R 4.0.2. The two-sided *P*-value <0.05 was considered to be statistically significant when comparing the two groups. To reduce the likelihood of type 1 error, the Bonferroni correction for multiple comparisons was performed in mixed linear models, and the threshold of *P*-value <0.008 (0.05/6) was considered to be statistically significant when comparing four groups.

## Supplementary Material

Supplementary Table 1
