# Supplementary material for: The joint effect of energy reduction with calcium supplementation on the risk factors of type 2 diabetes in the overweight population: a two-year randomized controlled trial
Source: Aging (Albany NY). 2021 Feb 11;13(4):5571–84. doi: 10.18632/aging.202485 (PMC7950291; doi:10.18632/aging.202485)
Supplement: Supplementary Table 1 [file aging-13-202485-s001.pdf]

SUPPLEMENTARY TABLE

Supplementary Table 1. Changes of dietary patterns in ERGs and NERGs.

Variables	ERGs			NERGs		
	Baseline	Follow-up	P-value	Baseline	Follow-up	P-value
Rice (g/d)	225 (165)	175 (110)	<0.001	215 (130)	225 (100)	0.053
Wheat (g/d)	150 (120)	125 (80)	<0.001	140 (110)	165 (95)	<0.001
Potato (g/d)	55 (60)	50 (50)	0.009	70 (95)	75 (90)	<0.001
Beans (g/d)	55 (65)	50 (55)	0.033	50 (60)	55 (55)	0.032
Vegetable (g/d)	280 (255)	300 (245)	<0.001	240 (130)	260 (210)	0.079
Fruit (g/d)	145 (155)	175 (145)	<0.001	125 (80)	135 (130)	0.090
Livestock (g/d)	80 (80)	40 (45)	<0.001	90 (90)	95 (90)	<0.001
Poultry (g /d)	30 (55)	25 (35)	<0.001	35 (50)	40 (55)	<0.001
Eggs (per d)	1.0 (1.4)	0.8 (0.6)	0.006	0.9 (0.8)	0.8 (0.6)	0.223
Milk (g/d)	100 (115)	55 (50)	<0.001	85 (95)	80 (75)	0.220
Fish (g /d)	35 (30)	25 (20)	0.103	30 (50)	40 (65)	0.004
Snack (g /d)	15 (30)	10 (20)	0.010	15 (35)	10 (15)	0.013
Energy (kcal/d)	2165 (984)	1673 (650)	<0.001	2076 (774)	2182 (666)	<0.001
Carbohydrate (g/d)	376 (169)	308 (103)	<0.001	366 (128)	375 (100)	<0.001
Protein (g/d)	76 (44)	54 (36)	<0.001	74 (34)	77 (33)	0.453
Lipid (g/d)	40 (28)	25 (20)	<0.001	41 (27)	41 (25)	0.932

Note: Data are presented as mean (Standard deviation).
